# Diagnostic Performance of Preoperative Imaging in Endometrial Cancer

**DOI:** 10.3390/curroncol30090597

**Published:** 2023-09-06

**Authors:** Chiaki Hashimoto, Shogo Shigeta, Muneaki Shimada, Yusuke Shibuya, Masumi Ishibashi, Sakiko Kageyama, Tomomi Sato, Hideki Tokunaga, Kei Takase, Nobuo Yaegashi

**Affiliations:** 1 Department of Obstetrics and Gynecology, Tohoku University School of Medicine, 1-1 Seiryo-machi, Aoba-ku, Sendai 980-8574, Japan; chiaki.hashimoto.a3@tohoku.ac.jp (C.H.);; 2 Advanced Research Center for Innovations in Next-Generation Medicine, Tohoku University, 2-1 Seiryo-machi, Aoba-ku, Sendai 980-8573, Japan; 3 Tohoku Medical Megabank Organization, Tohoku University, 2-1 Seiryo-machi, Aoba-ku, Sendai 980-8573, Japan; 4 Department of Diagnostic Radiology, Tohoku University Graduate School of Medicine, 1-1 Seiryo-machi, Aoba-ku, Sendai 980-8574, Japan

**Keywords:** endometrial cancer, computed tomography, magnetic resonance imaging, cancer stage, lymph node metastasis

## Abstract

Background: Endometrial cancer is one of the most common gynecological malignancies. Because the findings mentioned in radiogram interpretation reports issued by diagnostic radiologists influence treatment strategies, we aimed to evaluate the diagnostic accuracy of preoperative computed tomography (CT) and magnetic resonance imaging (MRI) interpretation results in clinically relevant settings. Methods: The clinical records of patients diagnosed with endometrial cancer treated at Tohoku University Hospital from January 2012 to December 2021 were reviewed. The preoperative and pathologically estimated cancer stages were compared based on the results mentioned in the radiogram interpretation report. Results: The preoperative and postoperative cancer stages were concordant in 70.0% of the patients. By contrast, the cancer stage was underdiagnosed and overdiagnosed in 21.7% and 8.2% of the patients, respectively. The sensitivities of MRI for deep myometrial invasion, cervical stromal invasion, vaginal invasion, and adnexal metastasis were 65.1%, 58.2%, 33.3%, and 18.4%, respectively. The sensitivity and specificity for pelvic lymph node metastasis using a combination of CT and MRI were 40.9% and 98.4%, respectively. Those for para-aortic lymph node metastases using CT were 37.0% and 99.5%, respectively. Conclusions: The low sensitivity observed in this study clarified the limitations of preoperative diagnostic performance in current clinical practice.

## 1. Introduction

Endometrial cancer is one of the most common malignancies of the female genital tract in Japan and other developed countries [1,2,3]. According to statistics from the National Cancer Institute’s Cancer Information Service, 17,780 patients were newly diagnosed with endometrial cancer in 2019, and 2644 died from the disease in 2020 in Japan [1].

The guidelines published by the Japanese Society of Gynecologic Oncology (JSGO) recommend multidisciplinary treatment with surgery and chemotherapy for endometrial cancer [4]. The JSGO treatment guidelines recommend hysterectomy, bilateral salpingo-oophorectomy, and regional lymphadenectomy as the basic surgical procedures for endometrial cancer, and adjuvant chemotherapy is recommended for patients with an intermediate or high risk of recurrence.

Randomized clinical trials in Europe and the United States have demonstrated the validity of minimally invasive surgeries (MISs), such as laparoscopic and robot-assisted surgeries, for early-stage endometrial cancer [5,6]. Upon receiving the results, laparoscopic and robot-assisted surgeries for early-stage endometrial cancer were covered by public medical insurance in Japan in 2014 and 2018, respectively. Additionally, the latest Japanese guidelines for treating endometrial cancer, published in 2023, propose omitting lymphadenectomy in patients preoperatively diagnosed with node-negative stage IA endometrial cancer with endometrioid grade 1 or 2 histology. Thus, preoperative diagnostic accuracy is becoming increasingly important for determining the appropriate surgical approach, especially for treating early-stage endometrial cancer. Even beyond early-stage endometrial cancer, the type of hysterectomy and area of lymphadenectomy are commonly determined based on a careful preoperative clinical image evaluation.

Preoperative clinical imaging is the standard method for estimating cancer stage. Computed tomography (CT) and magnetic resonance imaging (MRI) are recommended for preoperative evaluation per the current Japanese guidelines for the treatment of endometrial cancer and the Japanese guidelines for clinical imaging diagnosis [4,7,8]. Although positron emission tomography (PET)-CT is also an option, it is not recommended for routine evaluation, as not all patients have access to it.

In clinical practice, diagnostic radiologists issue radiogram interpretation reports for clinical imaging. In addition to the direct inspection of clinical imaging data, gynecological oncologists carefully refer to these interpretation reports when planning therapeutic strategies, including surgical approaches and procedures, as expert knowledge is necessary to appropriately interpret clinical imaging. Thus, these interpretation reports, together with other clinicopathological factors, significantly contribute to physicians’ decision making in treating endometrial cancer. In this study, we aimed to evaluate the diagnostic accuracy of radiogram interpretation results in endometrial cancer by comparing the preoperative cancer stage estimated using clinically relevant interpretation reports and pathologically confirmed findings.

## 2. Materials and Methods

The clinical information of patients with epithelial endometrial cancer treated at Tohoku University Hospital from January 2012 to December 2021 was retrospectively reviewed. This study followed the Declaration of Helsinki and was approved by the Institutional Review Board of Tohoku University School of Medicine (approval number: 2022-1-1163). As a basic policy, all patients diagnosed with endometrial cancer at Tohoku University Hospital underwent CT and MRI scans unless there were contraindications. Patients who underwent both preoperative systemic CT and pelvic MRI with or without contrast agents and were primarily treated with surgery, including regional lymphadenectomy or regional lymph node biopsy, were eligible for this study. Clinical imaging had to meet the following criteria for inclusion: (i) a CT scan slice ≤ 5 mm; (ii) a CT scan covering the thorax, abdomen, and pelvis; and (iii) an MRI sequence containing T2- and/or diffusion-weighted imaging. Patients treated with neoadjuvant chemotherapy, diagnosed with synchronous double cancer, with insufficient clinical or pathological data, or without radiogram interpretation reports for CT or MRI were excluded. Patients with non-epithelial cancer, such as leiomyosarcoma or endometrial stromal sarcoma, were also excluded. Patients with endometrial carcinosarcoma histology were not excluded from this study, as it is considered a unique histologic subtype of epithelial endometrial cancer and is treated in the same way as other epithelial endometrial cancers. Patients who underwent CT and/or MRI outside Tohoku University Hospital were not excluded if their imaging data met the criteria mentioned above.

Clinical information included age; clinically estimated and pathologically confirmed stages; histological subtype; details of surgical procedures, such as operative procedures; and the area of lymphadenectomy. Low-grade histology was defined as grade 1 or grade 2 endometrioid or mucinous histology. High-grade histology was defined as grade 3 endometrioid, serous, clear-cell, or other carcinoma histologies, according to the literature [9,10,11].

Because this study aimed to evaluate clinically relevant radiogram interpretation results, preoperative factors associated with cancer staging, such as myometrial invasion, cervical stromal invasion, vaginal invasion, adnexal metastasis, and distant metastasis, were fundamentally assessed based on the context described in the interpretation reports. The depth of myometrial invasion, the presence or absence of cervical stromal invasion, adnexal metastasis, and vaginal invasion were assessed based on an MRI report. Regional para-aortic node and distant metastases, including extra-regional lymph node metastases, were assessed using systemic CT. The pelvic lymph nodes were evaluated using computed reports of both CT and MRI.

In cases where tumor invasion or metastasis was described as “suspected”, we considered those as negative to avoid overdiagnosis in accordance with the basic principle in clinical practice. An exception was lymph node metastasis. If lymph node metastasis was described as “suspected” in the interpretation report, the original image was reviewed by the authors, and the short axis of the concerned lymph node was measured. Lymph node metastasis was considered positive if the short-axis diameter was ≥10 mm, complying with the Response Evaluation Criteria In Solid Tumors version 1.1 [12].

The preoperatively estimated International Federation of Gynecology and Obstetrics (FIGO) 2008 stage via clinical imaging (hereafter clinical FIGO stage) was determined based on the CT and MRI information, as described above [13]. As PET-CT was performed on a limited number of patients at the physician’s discretion, the results of PET-CT were collected but not used to determine the preoperative cancer stage in this study. The significance of PET-CT was independently addressed as a complementary analysis.

Clinicopathological parameters were compared using the Chi-squared test for categorical variables. Statistical significance was set at a two-sided *p*-value < 0.05. Clinical parameters are expressed as mean ± standard deviation or median with the range. Frequencies are expressed as numbers and percentages. All statistical analyses were performed using JMP^®^ Pro 16.0.0.

## 3. Results

### 3.1. Patient Characteristics

**Figure 1:**
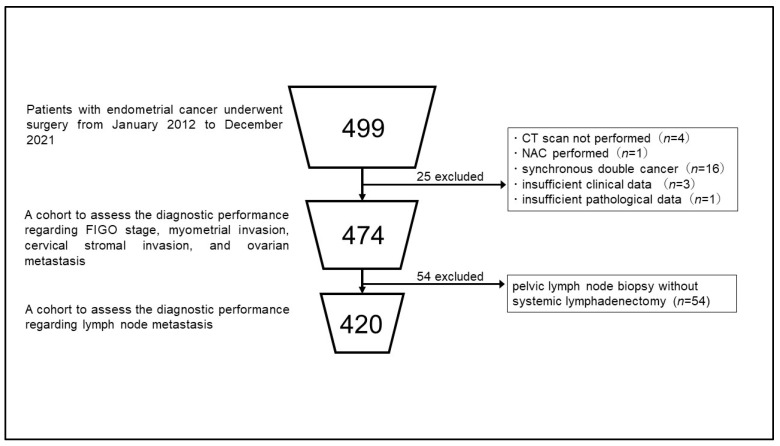
Scheme of patient selection. The results of patient selection and the cohorts analyzed in this study are summarized in the figure. Abbreviations: CT, computed tomography; NAC, neoadjuvant chemotherapy; FIGO, International Federation of Gynecology and Obstetrics.

In total, 474 patients were included in this study. A patient selection flowchart is shown in Figure 1. The patient characteristics are presented in Table 1.

As mentioned in the inclusion criteria, all the patients underwent preoperative systemic CT and pelvic MRI. The median time interval between the acquisition of clinical images and surgery was 48 days for CT (range, 1–137) and 51 days for MRI (range, 1–161). In addition, 37 patients underwent preoperative PET-CT. Regarding histological subtypes, 320 and 154 patients presented with low- and high-grade histologies, respectively. Of the 474 patients, 215 underwent para-aortic and pelvic lymphadenectomy. The remaining patients underwent pelvic lymphadenectomy or pelvic lymph node biopsy, as summarized in Table 1. At our institution, para-aortic lymphadenectomy is usually considered for patients with suspected para-aortic lymph node metastasis on clinical imaging, or for patients presenting with high-risk histology.

### 3.2. Clinically and Pathologically Confirmed International Federation of Gynecology and Obstetrics Stages

The clinical FIGO stage was concordant with the pathologically confirmed FIGO stage in 332 (70.0%) patients. By contrast, 103 (21.7%) and 39 (8.2%) patients were underdiagnosed and overdiagnosed, respectively, using preoperative CT and MRI (Table 2).

When stratified by histological subtype, the concordance, underdiagnosis, and overdiagnosis rates for low-grade histology were 74.1%, 16.9%, and 9.1%, respectively. By contrast, those for high-grade histology were 61.7%, 31.8%, and 6.5%, respectively (Supplementary Table S1). The Chi-squared test suggested a significant difference in the concordance rate between low- and high-grade histologies (*p* = 0.001).

### 3.3. Uterine Myometrial and Cervical Stromal Invasion

Regarding myometrial invasion, MRI indicated myometrial invasion < 1/2 in 338 (71.3%) patients and myometrial invasion ≥ 1/2 in 136 (28.7%) patients. By contrast, 323 and 151 patients were pathologically confirmed to have myometrial invasion < 1/2 and myometrial invasion ≥ 1/2, respectively. The sensitivity and specificity of MRI in detecting deep myometrial invasion ≥ 1/2 were 65.6% and 88.5%, respectively (Table 3).

The concordance, underdiagnosis, and overdiagnosis rates are summarized in Supplementary Table S2. As a lower concordance rate was observed among patients presenting with high-grade histology in the FIGO stage (Table 2), we next compared the sensitivity, specificity, and concordance rate of myometrial invasion according to histological subtype. Although the sensitivity and specificity for deep myometrial invasion ≥ 1/2 did not drastically differ, the concordance rate significantly differed between patients with low- and high-grade histologies (*p* = 0.0211; Table 3 and Supplementary Table S3). Interestingly, the positive prediction value in low-grade histology was lower than that in high-grade histology (62.9% vs. 83.3%), whereas the negative prediction value was higher in low-grade histology (89.6% vs. 70.5%). 

As several reports have indicated a poor diagnostic accuracy for myometrial invasion if the tumor is located at the lateral angle of the uterus [14,15,16], we further compared the diagnostic value of MRI between patients who were positive and negative for lesions at the lateral angle of the uterus. Based on a review of T2- and diffusion-weighted MRI sequences, 167 patients were considered to have tumors located at the lateral angle of the uterus. Although the sensitivity in these 167 patients was similar to that in the patients negative for lesions at the lateral angle of the uterus (66.2% vs. 65.1%), the specificity was lower in the 167 patients positive for lesions at the lateral angle of the uterus than that in the patients negative for lesions at the lateral angle of the uterus (76.2% vs. 92.9%; Table 3). The concordance rates also differed between the two groups (Supplementary Table S2).

The presence of leiomyoma or adenomyosis in the uterine corpus was also reported to influence diagnostic accuracy for myometrial invasion [17]. To assess the influence of leiomyoma and adenomyosis, we compared the diagnostic value of MRI between patients who had and did not have these diseases. As shown in Table 3 and Supplementary Tables S2, the concordance rate did not statistically differ, with a similar sensitivity between the two groups. 

The cervical stromal invasion was judged as positive based on MRI in 48 patients. Pathological examination confirmed cervical stromal invasion in 55 patients. The sensitivity and specificity were 58.2% and 96.2%, respectively (Supplementary Table S3).

### 3.4. Adnexal Metastasis and Vaginal Invasion

Adnexal metastasis and vaginal invasion were involved in the FIGO 2008 staging criteria. In this study, adnexal metastasis was pathologically confirmed in 38 (8.0%) patients. By contrast, MRI indicated adnexal metastasis in only eight patients. Of the eight patients, one was postoperatively diagnosed with thecoma. The remaining seven patients had adnexal metastasis. Although the specificity was 99.8%, the sensitivity was 18.4% (Supplementary Table S4).

Vaginal invasion was pathologically confirmed in six (1.3%) patients. By contrast, vaginal invasion was observed in two patients using MRI. The sensitivity and specificity were 33.3% and 100%, respectively (Supplementary Table S5).

### 3.5. Clinically and Pathologically Confirmed T Classification

Myometrial invasion, cervical stromal invasion, adnexal metastasis, and vaginal invasion were the major T-associated factors. Altogether, the concordant rate between the clinical and pathologically confirmed T classifications was 73.8%. By contrast, 16.9% and 9.3% of the patients were underdiagnosed and overdiagnosed, respectively, using preoperative MRI (Supplementary Table S6).

### 3.6. Pelvic Lymph Node Metastasis

For the subsequent assessment of lymph node metastasis, 54 patients who underwent lymph node biopsy were excluded to minimize bias, as these patients did not undergo a systemic pathological lymph node examination (Figure 1). In the remaining 420 patients, pelvic lymph node metastasis was preoperatively judged as positive in 24 patients based on CT and/or MRI, whereas lymph node metastasis was pathologically confirmed in 44 patients. Of the 24 patients who were preoperatively considered as positive for pelvic lymph node metastasis, 18 patients had pathologically confirmed lymph node metastasis. The sensitivity and specificity of the combination of CT and MRI for pelvic lymph node detection were 42.6% and 98.4%, respectively (Table 4).

Among the 26 patients who were preoperatively considered negative but pathologically positive for lymph node metastasis, four underwent PET-CT. Of the four patients, two had positive lymph node metastasis based on PET-CT.

### 3.7. Para-Aortic Lymph Node Metastasis

Of the 215 patients who underwent para-aortic lymph node dissection or biopsy, 27 had para-aortic lymph node metastasis. Preoperative CT suggested lymph node metastasis in 11 patients, and lymph node metastasis was pathologically confirmed in 10 of the 11 patients. The sensitivity and specificity were 37.0% and 99.5%, respectively (Table 5).

Among the 17 patients in whom lymph node metastasis was not preoperatively indicated but was detected via pathological examination, PET-CT was performed in 2 patients. PET-CT indicated lymph node metastasis in one of the two patients.

## 4. Discussion

In the current study, we examined the diagnostic performance of MRI and CT to estimate endometrial cancer stage, focusing on T and N classifications in clinically relevant settings. Our findings clarified a certain limitation in the accurate preoperative diagnosis of endometrial cancer stage using CT or MRI.

Preoperative diagnosis is closely associated with treatment strategies for endometrial cancer. As briefly mentioned in the Section 1, MISs, such as laparoscopic or robot-assisted surgery, are now widely used in Japan for patients with clinically diagnosed early-stage endometrial cancer. In addition, omitting regional lymphadenectomy is proposed in the latest guidelines for patients preoperatively diagnosed with stage IA endometrial cancer with low-grade histology. From a therapeutic perspective, underdiagnosis may increase the risk of recurrence by overlooking microscopic lymph node metastasis, which can result in the inappropriate omission of adjuvant therapy. However, overdiagnosis may impair the opportunity for patients to undergo minimally invasive surgery. 

As mentioned in the Introduction, the latest guidelines for treating endometrial cancer recommend MRI as a preoperative examination for evaluating local tumor extension. Several meta-analyses have evaluated the diagnostic value of MRI for myometrial invasion [18,19]. One meta-analysis reported that the pooled sensitivity and specificity of MRI were 83% and 82% [18], while another meta-analysis reported them to be 79% and 81%, respectively [19]. In our study, compared with these meta-analyses, the specificity was higher, but the sensitivity was lower, indicating that underestimation is more likely to occur in clinical practice than in clinical studies. Importantly, the latter meta-analysis also suggested better diagnostic performance of MRI in patients aged <60 years [19]. As endometrial cancer with high-grade histology is more frequently observed in older patients [3], the meta-analysis indicates the difficulty in conducting an accurate preoperative evaluation of myometrial invasion in patients presenting with high-grade histology. Our results also indicate that myometrial invasion tended to be underdiagnosed among patients presenting with high-grade histology. High-grade endometrial cancer is known to present with nonconsecutive invasion of the uterine myometrium, which may be a possible reason for the diagnostic difficulty in myometrial invasion among patients with endometrial carcinoma with high-risk histology [20].

Cervical stromal invasion is an important factor in determining the hysterectomy type. Although the current guidelines for the treatment of endometrial cancer suggest extrafacial or extended simple hysterectomy if the cervical stromal invasion is preoperatively estimated, radical hysterectomy or modified radical hysterectomy is also mentioned as a possible option because of the inconsistency between preoperative and postoperative diagnoses. In accordance with the guideline documentation, a meta-analysis reported the sensitivity and specificity of MRI as 53% and 95%, respectively, which are similar to the results of this study [19]. Altogether, one of the limitations of MRI is low sensitivity in the preoperative diagnosis of cervical stromal invasion.

As an alternative, a recent study reported the diagnostic performance of a transvaginal ultrasound in evaluating myometrial and cervical stromal invasion [21]. Diagnostic performance was similar between the ultrasound and MRI in assessing myometrial invasion. However, the transvaginal ultrasound was superior to MRI in assessing cervical stromal invasion. Transvaginal ultrasound findings may be informative in cases where cervical stromal invasion is not ruled out by MRI findings, or in patients with contraindication for MRI.

The sensitivity of MRI to detect ovarian metastasis was <20% in our study. Although the sensitivity reported in a previous study was higher than that in our study at 51.7%, it still indicated that approximately half of the ovarian metastases were undetectable via MRI. Although bilateral salpingo-oophorectomy is the standard surgical strategy for treating endometrial cancer, both the National Comprehensive Cancer Network and Japanese guidelines mention the possibility of ovarian preservation in young patients under certain conditions [4,22]. As the accurate preoperative exclusion of adnexal metastasis is essential for ovarian preservation, a careful inspection using MRI, transvaginal sonography, and/or PET-CT is considered necessary.

Lymph node metastasis is another important issue to consider when planning surgical strategies for endometrial cancer. In a meta-analysis of the diagnostic performance of CT and MRI for lymph node metastasis, the mean sensitivity and specificity of CT were 45% and 88% compared with 72% and 97%, respectively, for MRI [23]. Although the diagnostic performance of CT and MRI reported in previous studies varied [19,24,25], the reported sensitivity was not satisfactory for predicting lymph node metastasis using a single imaging modality. Thus, the integration of MRI and CT results is necessary to increase diagnostic accuracy, particularly for pelvic lymph node evaluation. However, the sensitivity for pelvic lymph node metastasis was approximately 40%, even with the integration of CT and MRI in this study. Our results indicate that the omission of lymphadenectomy should be carefully determined by simultaneously considering clinical imaging results and other risk factors of recurrence.

Importantly, most risk factors of recurrence are confirmed pathologically in endometrial cancer. By contrast, several studies reported an association between the preoperatively estimated tumor volume and progression-free survival [26,27]. Although it remains controversial whether the preoperatively calculated tumor volume is an independent risk factor for recurrence [28], preoperatively evaluated tumor volume may be informative in assessing the risk of recurrence in addition to pathological findings.

The low sensitivity of CT and MRI for lymph node metastasis also raises the possibility of adding PET-CT to CT/MRI to increase sensitivity. We did not assess the significance of PET-CT, which is a limitation of our study, because (i) <10% of the patients underwent preoperative PET-CT scans, and (ii) it was not used to investigate lymph node metastasis in a substantial number of patients. Although PET-CT has been reported to be useful for evaluating lymph node metastasis [25,29,30], several studies have highlighted its low sensitivity in detecting lymph node metastasis [31,32,33]. Its cost is also a drawback. In Japan, the cost of a single PET-CT scan is approximately three to four times as expensive as that of contrast-enhanced CT. Considering the reported sensitivity of PET-CT in comparison with the sensitivity of CT and/or MRI in this study for the detection of lymph node metastasis, the addition of routine preoperative PET-CT to CT and MRI for every patient is not a realistic strategy in terms of cost-effectiveness. Accessibility and increased radiation exposure are also essential concerns when performing PET-CT in addition to CT. However, further studies are warranted to determine the significance and appropriate stratification of PET-CT in the preoperative evaluation of endometrial cancer.

The time interval between clinical imaging and surgery is an important issue when considering the preoperative diagnostic accuracy. The delay of surgery after the acquisition of clinical imaging may increase the frequency of preoperative underdiagnosis. In large clinical trials, the interval between clinical imaging acquisition to surgery was set at a maximum of 2 weeks to assess the diagnostic accuracy of clinical imaging in gynecologic malignancies [25,34,35]. The time interval in this study might be one of the reasons for the diagnostic performance being lower than that in previous studies. However, it is not always possible to perform surgery shortly after clinical imaging evaluation in clinical practice. From this perspective, the diagnostic accuracy found in this study may be more practical.

As one of the “-omics”, radiomics is gaining attention in the fields of precision medicine, prognostic biomarkers, and differential diagnosis [36]. By contrast with traditional radiogram interpretation, radiomics is based on quantitative data determined via a comprehensive clinical image analysis focusing on parameters of interest, such as the shape, intensity, and texture of the target lesion [37]. Regarding preoperative endometrial cancer staging, a systemic review reported the potential benefit of radiomics of pelvic MRI for the preoperative estimation of deep myometrial invasion, lymphovascular space invasion, tumor grading, and lymph node metastasis [38]. Additionally, several studies reported the possible advantage of integrating radiomics into preoperative diagnosis for an accurate estimation of lymph node involvement in endometrial cancer [39,40,41]. Further investigations focusing on radiomics in combination with multi-omics analyses or artificial intelligence technology are warranted.

Unlike general diagnostic radiology studies, our study was not designed as a retrospective imaging review. The type of MRI sequencing, the thickness of the CT slice, the use of contrast agents, and imaging devices varied among the enrolled patients. These are major limitations of this study in terms of scientifically assessing the diagnostic performance. However, it is difficult to unify all factors associated with imaging in clinical practice. More importantly, it is not retrospective imaging review results but interpretation results by general diagnostic radiologists that influence the treatment plan for patients. Therefore, we believe that our findings are relevant in actual clinical settings, which we consider to be a strength. In addition, the cohort size was relatively large with more than 400 patients, which is another advantage of this study. We consider the result of this study valuable for elucidating the current problem of clinical imaging in clinical practice.

In conclusion, we retrospectively reviewed the diagnostic performance of CT and MRI in the preoperative evaluation of endometrial cancer. We found a low sensitivity of MRI, CT, or the combination of both in detecting multiple parameters regarding endometrial cancer stages, such as deep myometrial invasion, cervical stromal invasion, ovarian metastasis, vaginal invasion, and lymph node metastasis. Indications for surgical procedures, including lymphadenectomy, should be carefully determined based on clinical imaging and an assessment of risk factors. Preoperative imaging evaluation in clinical practice should be further optimized, including indications for PET-CT.

## Figures and Tables

**Table 1:** Patient characteristics.

	Median (Range)	
Age	56.5 (21–77)	
	No. of Patients	%
FIGO 2008 stage		
IA	280	59.1
IB	76	16
II	28	5.9
IIIA	18	3.8
IIIB	5	1.1
IIIC1	25	5.3
IIIC2	22	4.6
IVA	1	0.2
IVB	19	4
Histology		
Endometrioid grades 1–2	317	66.9
Endometrioid grade 3	53	11.2
Mucinous	3	0.6
Serous	43	9.1
Clear cell	12	2.5
Mixed	28	5.9
Carcinosarcoma	16	3.4
Undifferentiated or dedifferentiated	2	0.4
Surgical approach		
Laparotomy	317	66.9
Laparoscopy or robot-assisted surgery	157	33.1
Lymphadenectomy		
PLN	205	43.2
PLN and PALN	215	45.4
Biopsy	54	11.4

Abbreviations: FIGO, International Federation of Gynecology and Obstetrics; PLN, pelvic lymph node; PALN, para-aortic lymph node; No., number.

**Table 2:** Concordance rate between clinically estimated and pathologically confirmed FIGO stages.

	Overdiagnosed		Concordant		Underdiagnosed	
FIGO 2008 Stage	No. of Patients	(%)	No. of Patients	(%)	No. of Patients	(%)
IA	NA	NA	247	(80.2)	61	(19.8)
IB	23	(26.1)	41	(46.6)	24	(27.3)
II	8	(24.2)	19	(57.6)	6	(18.2)
IIIA	1	(8.3)	6	(50.0)	5	(41.7)
IIIB	0	(0)	2	(100)	0	(0)
IIIC1	4	(23.5)	7	(41.2)	6	(35.3)
IIIC2	1	(11.1)	7	(77.8)	1	(11.1)
IVA	0	(0)	0	(0)	0	(0)
IVB	2	(40.0)	3	(60.0)	NA	NA
Total	39	(8.2)	332	(70.0)	103	(21.7)

Abbreviations: NA, not applicable.

**Table 3:** Diagnostic performance of MRI in myometrial invasion ≥ 1/2.

All Patients			
	Pathological diagnosis		
Preoperative diagnosis	Positive	Negative	
Positive	99	37	PPV
			72.8%
Negative	52	286	NPV
			84.6%
	Sensitivity	Specificity	Accuracy
	65.6%	88.5%	81.2%
Low-Grade Histology			
	Pathological diagnosis		
Preoperative diagnosis	Positive	Negative	
Positive	44	26	PPV
			62.9%
Negative	26	224	NPV
			89.6%
	Sensitivity	Specificity	Accuracy
	62.9%	89.6%	83.8%
High-Grade Histology			
	Pathological diagnosis		
Preoperative diagnosis	Positive	Negative	
Positive	55	11	PPV
			83.3%
Negative	26	62	NPV
			70.5%
	Sensitivity	Specificity	Accuracy
	67.9%	84.9%	76.0%
Tumor without the Lateral Angle of the Uterus			
	Pathological diagnosis		
Preoperative diagnosis	Positive	Negative	
Positive	45	17	PPV
			72.6%
Negative	23	222	NPV
			90.6%
	Sensitivity	Specificity	Accuracy
	66.2%	92.9%	87.0%
Tumor Located at the Lateral Angle of the Uterus			
	Pathological diagnosis		
Preoperative diagnosis	Positive	Negative	
Positive	54	20	PPV
			73.0%
Negative	29	64	NPV
			68.8%
	Sensitivity	Specificity	Accuracy
	65.1%	76.2%	70.7%
Leiomyoma and/or Adenomyosis Absent			
	Pathological diagnosis		
Preoperative diagnosis	Positive	Negative	
Positive	62	18	PPV
			77.5%
Negative	31	187	NPV
			85.8%
	Sensitivity	Specificity	Accuracy
	66.7%	91.2%	83.6%
Leiomyoma and/or Adenomyosis Present			
Deep myometrial invasion	Pathological diagnosis		
Preoperative diagnosis	Positive	Negative	
Positive	37	19	PPV
			66.1%
Negative	21	99	NPV
			82.5%
	Sensitivity	Specificity	Accuracy
	63.8%	83.9%	77.3%

Abbreviations: PPV, positive prediction value; NPV, negative prediction value.

**Table 4:** Diagnostic performance of CT/NRI in pelvic lymph node metastasis.

	Pathological Diagnosis		
Preoperative Diagnosis	Positive	Negative	
Positive	18	6	PPV
			75.0%
Negative	26	370	NPV
			93.4%
	Sensitivity	Specificity	Accuracy
	40.9%	98.4%	92.4%

**Table 5:** Diagnostic performance of CT in para-aortic lymph node metastasis.

	Pathological Diagnosis		
Preoperative Diagnosis	Positive	Negative	
Positive	10	1	PPV
			90.9%
Negative	17	187	NPV
			91.7%
	Sensitivity	Specificity	Accuracy
	37.0%	99.5%	91.6%

## Data Availability

The data presented in this study are available on request from the corresponding author with reasonable reasons.

## Supplementary Materials

The following supporting information can be downloaded at https://www.mdpi.com/article/10.3390/curroncol30090597/s1. Table S1: Comparison of preoperative and postsurgical cancer stages; Table S2: Concordance rate between clinically estimated and pathologically confirmed myometrial invasion; Table S3: Diagnostic performance of MRI for cervical stromal invasion; Table S4: Diagnostic performance of MRI for adnexal metastasis; Table S5: Diagnostic performance of MRI for vaginal invasion; and Table S6: Diagnostic performance of MRI for T classification.
