# Supplementary material for: Diagnostic Performance of Preoperative Imaging in Endometrial Cancer
Source: Curr Oncol. 2023 Sep 6;30(9):8233–44. doi: 10.3390/curroncol30090597 (PMC10527880; doi:10.3390/curroncol30090597)
Supplement: Supplementary file 1 [file curroncol-30-00597-s001.zip › curroncol-2560202-supplementary.pdf]

Supplementary Information

Table S1. Comparison of preoperative and postsurgical cancer stages.

All cases

	Pathological stage									
Preoperative stage	IA	IB	II	IIIA	IIIB	IIIC1	IIIC2	IVA	IVB	Total
IA	**247**	33	6	7	2	7	3	0	3	308
IB	23	**41**	2	3	0	5	9	0	5	88
II	6	2	**19**	1	0	4	0	0	1	33
IIIA	1	0	0	**6**	0	2	0	0	3	12
IIIB	0	0	0	0	**2**	0	0	0	0	2
IIIC1	2	0	1	1	0	**7**	2	1	3	17
IIIC2	1	0	0	0	0	0	**7**	0	1	9
IVA	0	0	0	0	0	0	0	**0**	0	0
IVB	0	0	0	0	1	0	1	0	**3**	5
Total	280	76	28	18	5	25	22	1	19	474

Low-grade cases

	Pathological stage									
Preoperative stage	IA	IB	II	IIIA	IIIB	IIIC1	IIIC2	IVA	IVB	Total
IA	**199**	21	2	5	2	6	1	0	0	236
IB	18	**23**	0	0	0	4	5	0	0	50
II	4	2	**8**	1	0	2	0	0	0	17
IIIA	1	0	0	**3**	0	2	0	0	0	6
IIIB	0	0	0	0	**0**	0	0	0	0	0
IIIC1	2	0	0	0	0	**3**	1	0	2	8
IIIC2	1	0	0	0	0	0	**1**	0	0	2
IVA	0	0	0	0	0	0	0	**0**	0	0
IVB	0	0	0	0	0	0	1	0	**0**	1
Total	225	46	10	9	2	17	9	0	2	320

High-grade cases

	Pathological stage									
Preoperative stage	IA	IB	II	IIIA	IIIB	IIIC1	IIIC2	IVA	IVB	Total
IA	**48**	12	4	2	0	1	2	0	3	72
IB	5	**18**	2	3	0	1	4	0	5	38
II	2	0	**11**	0	0	2	0	0	1	16
IIIA	0	0	0	**3**	0	0	0	0	3	6
IIIB	0	0	0	0	**2**	0	0	0	0	2
IIIC1	0	0	1	1	0	**4**	1	1	1	9
IIIC2	0	0	0	0	0	0	**6**	0	1	7
IVA	0	0	0	0	0	0	0	**0**	0	0
IVB	0	0	0	0	1	0	0	0	**3**	4
Total	55	30	18	9	3	8	13	1	17	154

Table S2. Concordance rate between clinically estimated and pathologically confirmed myometrial invasion.

	**Overdiagnosed**	**Concordant**	**Underdiagnosed**	**p-value**
All cases	37 (7.8%)	385 (81.2%)	52 (11.0%)	
Low-grade histology	26 (8.1%)	268 (83.7%)	26 (8.1%)	0.0211
High-grade histology	11 (7.1%)	117 (76.0%)	26 (16.9%)	
Tumor without the lateral angle of the uterus	17 (5.5%)	267 (87.0%)	23 (7.5%)	0.0001
Tumor located at the lateral angle of the uterus	20 (12.0%)	118 (70.7%)	29 (17.4%)	
Leiomyoma or adenomyosis absent	18 (6.0%)	249 (83.6%)	31 (10.4%)	0.14
Leiomyoma or adenomyosis present	19 (10.8%)	136 (77.3%)	21 (11.9%)	

Table S3. Diagnostic performance of MRI for cervical stromal invasion.

	Pathological diagnosis		
**Preoperative diagnosis**	**Positive**	**Negative**	
Positive	32	16	PPV 66.7%
Negative	23	403	NPV 94.6%
	Sensitivity 58.2%	Specificity 96.2%	Accuracy 91.8%

Abbreviations: PPV, positive predictive value; NPV, negative predictive value

Table S4. Diagnostic performance of MRI for adnexal metastasis.

Preoperative diagnosis	Pathological diagnosis Positive	Pathological diagnosis Negative	
Positive	7	1	PPV<br>87.5%
Negative	31	435	NPV<br>93.3%
	Sensitivity<br>18.4%	Specificity<br>99.8%	Accuracy<br>93.2%

Abbreviations: PPV, positive predictive value; NPV, negative predictive value; MRI, magnetic resonance imaging

Table S5. Diagnostic performance of MRI for vaginal invasion.

Preoperative diagnosis	Pathological diagnosis		
	Positive	Negative	
Positive	2	0	PPV<br>100%
Negative	4	468	NPV<br>99.2%
	Sensitivity<br>33.3%	Specificity<br>100%	Accuracy<br>99.2%

Abbreviations: PPV, positive predictive value; NPV, negative predictive value

Table S6. Diagnostic performance of MRI for T classification

Overdiagnosed	Concordant	Underdiagnosed
37 (7.8%)	385 (81.2%)	52 (11.0%)
